# Multi-Omics Characterization of Type 2 Diabetes Mellitus-Induced Cognitive Impairment in the db/db Mouse Model

**DOI:** 10.3390/molecules27061904

**Published:** 2022-03-15

**Authors:** Xiaoxuan Song, Zeyu Zhu, Xiaohang Qian, Xiaoli Liu, Shengdi Chen, Huidong Tang

**Affiliations:** 1 Department of Neurology and Institute of Neurology, Ruijin Hospital, Shanghai Jiao Tong University School of Medicine, Shanghai 200025, China; songxx@rjlab.cn (X.S.); zhuzy@rjlab.cn (Z.Z.); qianxh@rjlab.cn (X.Q.); 2 Department of Neurology, Shanghai Fengxian District Central Hospital, Shanghai Jiao Tong University Affiliated Sixth People’s Hospital South Campus, Shanghai 201400, China; liuxl@rjlab.cn; 3 Department of Neurology, Shanghai Guangci Memorial Hospital, Shanghai 200025, China

**Keywords:** type 2 diabetes mellitus, cognitive impairment, transcriptome, metabolome, gut microbiota

## Abstract

Type 2 diabetes mellitus (T2DM) is a complex metabolic disorder frequently accompanied by cognitive impairment. Contributing factors such as modern lifestyle, genetic predisposition, and gene environmental interactions have been postulated, but the pathogenesis remains unclear. In this study, we attempt to investigate the potential mechanisms and interventions underlying T2DM-induced cognitive deficits from the brain–gut axis perspective. A combined analysis of the brain transcriptome, plasma metabolome, and gut microbiota in db/db mice with cognitive decline was conducted. Transcriptome analysis identified 222 upregulated gene sets and 85 downregulated gene sets, mainly related to mitochondrial respiratory, glycolytic, and inflammation. In metabolomic analysis, a total of 75 significantly altered metabolites were identified, correlated with disturbances of glucose, lipid, bile acid, and steroid metabolism under disease state. Gut microbiota analysis suggested that the species abundance and diversity of db/db mice were significantly increased, with 23 significantly altered genus detected. Using the multi-omics integration, significant correlations among key genes (*n* = 33), metabolites (*n* = 41), and bacterial genera (*n* = 21) were identified. Our findings suggest that disturbed circulation and brain energy metabolism, especially mitochondrial-related disturbances, may contribute to cognitive impairment in db/db mice. This study provides novel insights into the functional interactions among the brain, circulating metabolites, and gut microbiota.

## 1. Introduction

Type 2 diabetes (T2DM) is a group of metabolic syndromes strongly influenced by a complex combination of metabolic, genetic, and environmental factors [1]. Although the pathogenesis of T2DM is inconclusive, it is highly associated with insufficient insulin secretion or insulin resistance (IR) of target organs [2]. Recently, increasing evidence has indicated that cognitive impairment is a severe complication and comorbidity of T2DM [3,4,5]. Pre-diabetes and T2DM could accelerate the progression from cognitive impairment to dementia [6,7]. Alzheimer’s disease (AD) is the major cause of dementia, accounting for 60 to 80% of all cases [8]. The risk of AD and other dementia in individuals with T2DM is 1.5 times that of individuals without T2DM [9]. Interestingly, AD has been considered by some as type 3 diabetes mellitus, a neurometabolic disorder [10,11,12]. Abnormal insulin signals, dysregulated glucose metabolism, and the formation of advanced glycation products are common etiologies of cognitive impairment due to T2DM, and these are also indicated as potential associations between T2DM and AD [13]. Despite shared pathological features, including insulin resistance and β-Amyloid (Aβ) deposition, it is still unclear what specific alterations in T2DM could be responsible for the increased susceptibility to AD [10]. With the advent of population aging, more than 50 million people suffer from dementia globally, which will increase to 152 million by 2050 [14]. It is conceivable that the increasing incidence of cognitive impairment and dementia are possibly on account of not only population aging but also the T2DM epidemic [15]. Therefore, exploring the risk factors and prevention mechanisms of cognitive dysfunction associated with T2DM will make a great difference.

Owing to the rapid development of high-throughput technologies and bioinformatics technologies, omics-based approaches (genomics, transcriptomics, metabolomics, etc.) have gained interest and brought great convenience to system biological research [16]. System biology as an interdisciplinary field of study focuses on complex interactions within biological systems, enabling researchers to better understand how molecules change in normal processes and disease status [17]. Based on metabolomics analysis, Kavanagh et al. discovered that peripheral glucose dysregulations in T2DM monkeys are coincident with alterations in cerebral metabolism and correlate with early amyloid deposition within the brain [18]. In recent years, studies have shown that gut microbiota is a key factor of most chronic diseases, including diabetes, Alzheimer’s disease, and Parkinson’s disease, etc. [19,20,21]. Microbiome analysis shows that altered composition of the gut microbiota is commonly observed in both AD and T2DM animal models, such as *Bacteroidetes*, *Actinobacteria,* or *Firmicutes* phyla, etc. [22]. In addition, human studies have also confirmed these observations, reporting a changed microbial characterization in the two diseases [20,23]. Growing evidence has indicated that gut microbiota not only could participate in the metabolism of the body [24] but could also regulate brain function through the brain–gut–microbiota axis [25], which provides a potential relevant link between cognitive impairment and metabolic dysregulations.

Although the single omics-based approach is able to investigate a specific type of molecules (genes, metabolites, and proteins, etc.) comprehensively, it cannot capture the synergistic interactions and complementary effects between multiple types of molecules [26]. In order to do so, the multi-omics approaches should be given priority, which helps to reveal the molecular interrelations and molecular dynamics at a disease state [26,27]. Therefore, to explore the potential mechanisms and interventions underlying T2DM-induced cognitive impairment, we conducted a combined analysis of the brain transcriptome, plasma metabolome, and gut microbiota in T2DM mice with cognitive decline.

## 2. Results

### 2.1. Cognitive Impairment in db/db Mice

**Figure 1:**
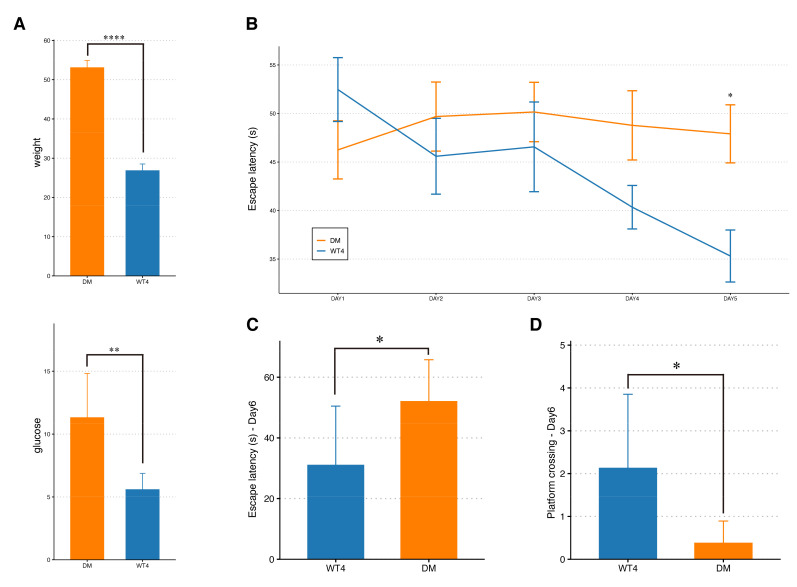
Body weight and blood glucose measurement and cognitive function assessment. (**A**) The body weight and blood glucose of 4-month-old db/db mice were significantly higher than those of wild-type (wt) mice (*t*-test; ** 0.001 < *p* < 0.01, **** *p* < 0.0001). (**B**) Line chart of average escape latency (Two-way ANOVA; * 0.01 < *p* < 0.05). (**C**,**D**) On day six, the average escape latency of db/db mice was significantly prolonged, while the number of platform crossing was significantly reduced (*t*-test; * 0.01 < *p* < 0.05).

Compared with wild-type (wt) mice, db/db mice showed significantly higher body weight and fasting blood glucose levels (Figure 1A). The Morris water maze (MWM) test illustrated a notable difference in cognitive performance between the db/db group and the wt group. During the training trials, escape latencies were significantly higher in the db/db group than in the wt group, especially on day 5 (Figure 1B). In the probe test, db/db mice spent significantly more time to reach the escape platform than in the wt group (Figure 1C). Moreover, the number of platform crossings was significantly reduced in the db/db group compared to the wt group (Figure 1D). Taken together, the tests demonstrated deficient spatial learning and memory in the db/db group relative to the wt group.

### 2.2. Transcriptomic Analysis

**Figure 2:**
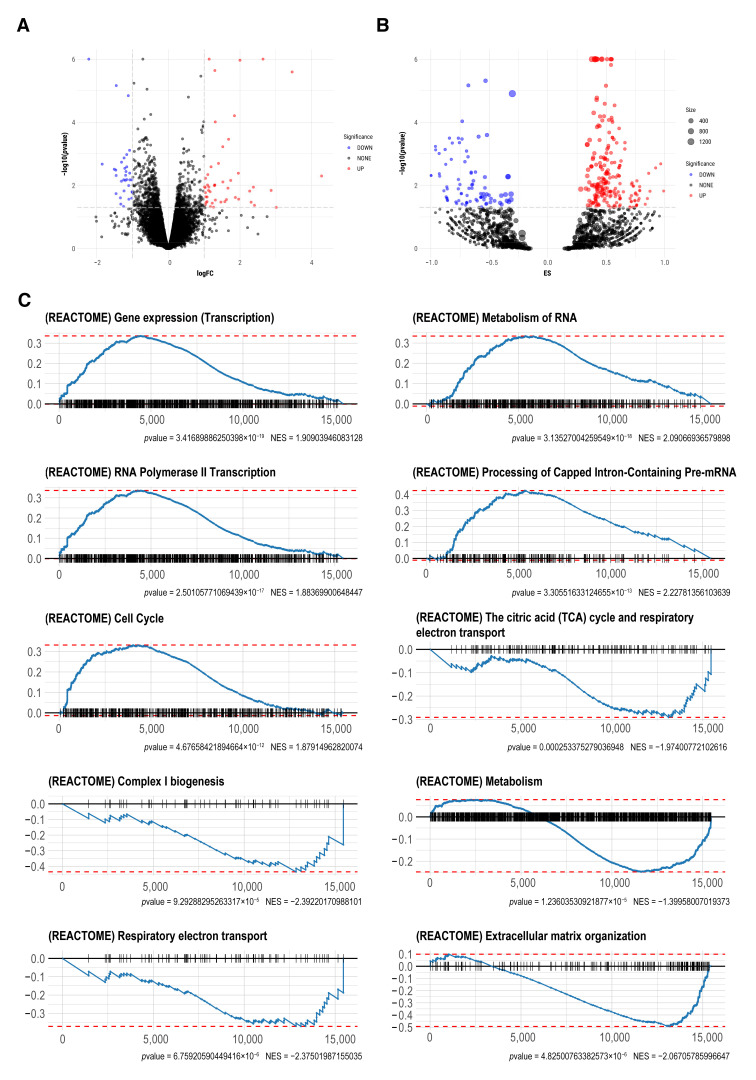
Differential gene expression analysis and GSEA. (**A**) Volcano plot of differentially expressed genes (DEGs). Red represents upregulated genes, blue represents downregulated genes, and black represents genes with no significant change in expression. (**B**) Volcano plot of gene set enrichment analysis (GSEA) based on the Reactome database. Red represents pathways mainly composed of upregulated genes, blue represents pathways mainly composed of downregulated genes, and black represents pathways with no significant change in gene expression. The size of dots represents the count of genes in each pathway. For visualization purposes, −log10 (*p*-value) greater than 6 was defined as equal to 6. (**C**) Enrichment plots for top five upregulated and downregulated pathways based on the *p*-value.

The gene count data of db/db mice and wt mice are presented in Table S1. Based on the aforementioned threshold (|logFC| ≥ 1, *p* < 0.05), 80 genes (48 upregulated and 32 downregulated) were filtered as differentially expressed genes (DEGs) (Figure 2A). Gene set enrichment analysis (GSEA) was performed to detect which biological pathways were enriched in db/db mice, based on the Reactome database. Two hundred and twenty-two gene sets were identified as upregulated and 85 gene sets were downregulated (Figure 2B, Table 1). Among them, the top five gene sets were selected respectively based on the *P*-value ranking, to generate GSEA enrichment plots. As shown in Figure 2C, “Gene expression (transcription)”, “Metabolism of RNA”, “RNA polymerase II transcription”, “Processing of capped intron-containing pre-mRNA”, and “Cell cycle” had higher expression in db/db mice. “The citric acid (TCA) cycle and respiratory electron transport”, “Complex I biogenesis”, “Metabolism”, “Respiratory electron transport”, and “Extracellular matrix organization” were downregulated in db/db mice (Figure 2C), indicating dysregulated mitochondrial metabolism in the brain of db/db mice.

### 2.3. Metabolomic Analysis

**Figure 3:**
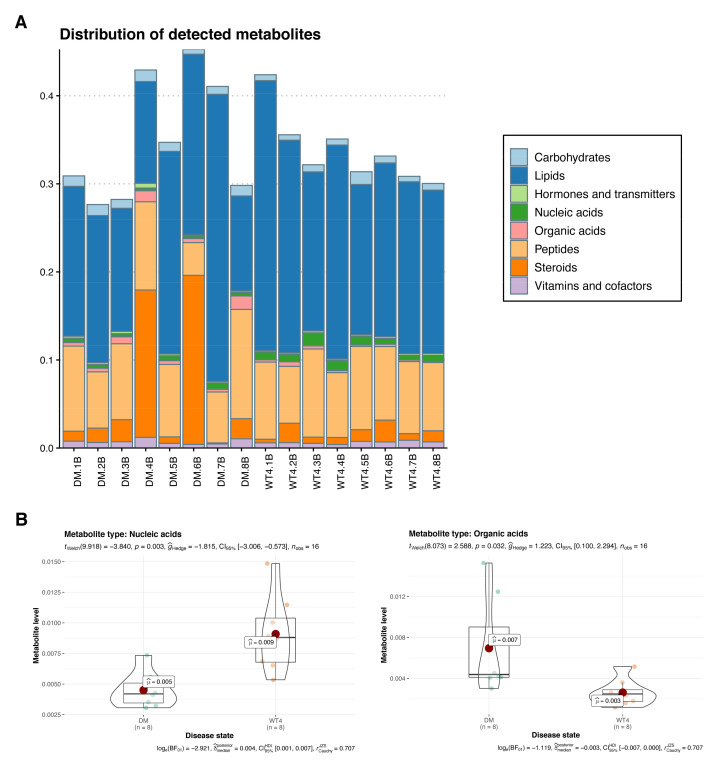
Metabolite abundance analysis. (**A**) Stacked bar graph of metabolite abundance. (**B**) Violin plots of nucleic acids abundance and organic acids abundance in two groups of mice.

To analyze the distribution of detected metabolites (Tables S2 and S3), metabolites were classified into carbohydrates, lipids, hormones and transmitters, nucleic acids, organic acids, peptides, steroids, vitamins, and cofactors based on the Kyoto Encyclopedia of Genes and Genomes (KEGG) database. As shown in Figure 3A, lipids accounted for the highest percentage, followed by peptides, with fewer hormones, transmitters, and organic acids. Compared to wt mice, nucleic acids in db/db mice were significantly decreased (*p* = 0.003), while organic acids were significantly increased (*p* = 0.032), suggesting metabolic disorders of nucleic acid and organic acid in the diseased group (Figure 3B).

**Figure 4:**
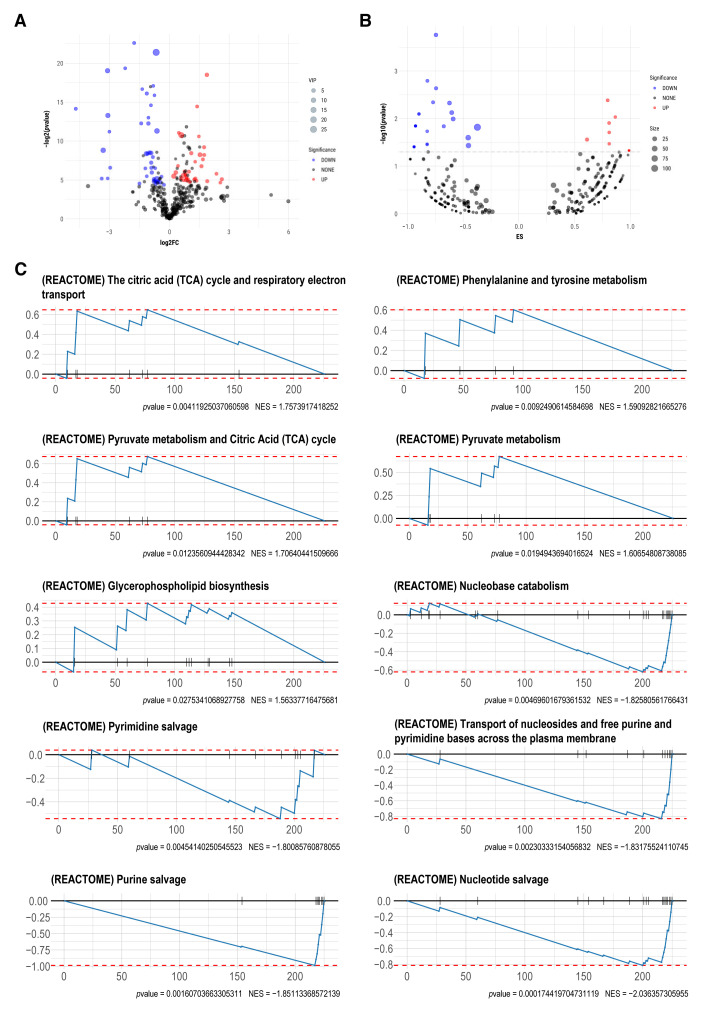
Differential metabolite analysis and MSEA. (**A**) Volcano plot of significantly different metabolites. Red represents a significant increase in abundance, green represents a significant decrease in abundance, and black represents no significant change in abundance. The size of dots represents the VIP of metabolites. (**B**) Volcano plot of metabolite set enrichment analysis (MSEA) on basis of the Reactome database. Red and blue represent pathways mainly enriched by metabolites with increased or decreased abundance, respectively. Black represents pathways enriched by metabolites with no significant alteration. For visualization purposes, −log10 (*p*-value) greater than 6 was defined as equal to 6. (**C**) Enrichment plots for top five pathways mainly composed of metabolites with increased or decreased abundance based on the *p*-value.

Based on the Student’s *t*-test and orthogonal partial least squares-discriminant analysis (OPLS-DA), a total of 75 significantly altered metabolites were identified, including 34 upregulated and 41 downregulated metabolites (Figure 4A). On the basis of the Reactome database, metabolite set enrichment analysis (MSEA) was further performed to detect pathways with significant alteration between two groups. Ten upregulated and 26 downregulated pathways were identified (Figure 4B, Table 2). Enrichment plots were generated for the top five pathways ranked based on the *p*-value. The results showed that significantly upregulated pathways included “The citric acid (TCA) cycle and respiratory electron transport”, “Phenylalanine and tyrosine metabolism”, “Pyruvate metabolism and Citric Acid (TCA) cycle”, “Pyruvate metabolism”, and “Glycerophospholipid biosynthesis”, and downregulated pathways consisted of “Nucleotide salvage”, “Purine salvage”, “Transport of nucleosides and free purine and pyrimidine bases across the plasma membrane”, “Pyrimidine salvage”, and “Nucleobase catabolism” (Figure 4C).

### 2.4. Gut Microbiota Analysis

**Figure 5:**
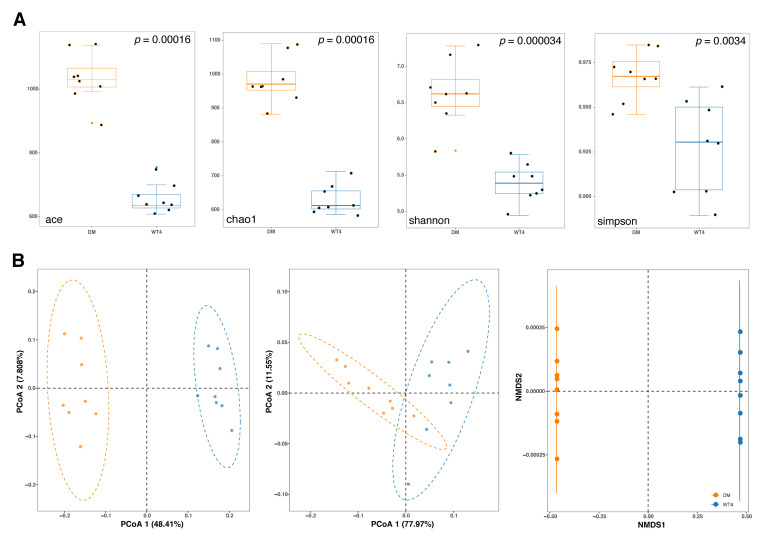
Bacterial diversity assessment. (**A**) Comparisons of α-diversity. The abundance-based coverage estimators (ACE), Chao1, Shannon, and Simpson indexes are displayed. (**B**) Principle coordinate analysis (PCoA) based on weighted or unweighted unifrac distance and NMDS are shown from left to right.

**Figure 6:**
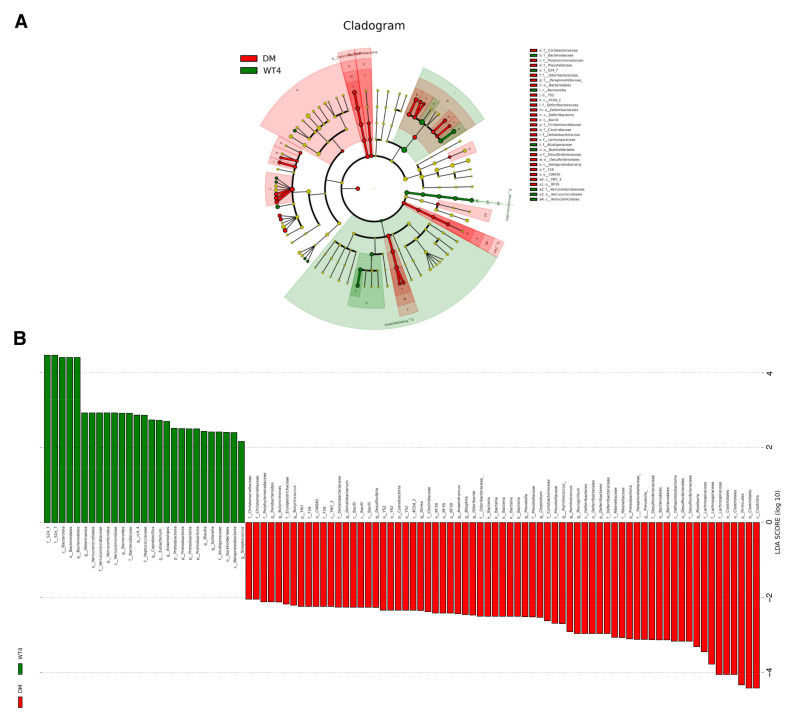
Taxonomic differences of fecal microbiota between db/db and WT mice. (**A**) A cladogram reporting the differences which were represented in the color of the most abundant class between the two groups of mice. Red indicates db/db mice, green wt mice, and yellow non-significant. The diameter of each circle is proportional to the relative taxon’s abundance. (**B**) The histogram of linear discriminant analysis (LDA) scores. The length of each bar represents the impact size of the bacterial taxa differentially abundant.

The observed operation taxonomy units (OTUs) were presented in Table S4. The abundance-based coverage estimators (ACE) and Chao1 were used to assess the abundance of gut microbiota, and the Shannon and Simpson index was used to assess species diversity. As shown in Figure 5A, all the four indexes of the disease group were significantly higher than the control, suggesting that the species abundance and diversity of db/db mice were significantly increased. Principle coordinate analysis (PCoA) based on both weighted and unweighted unifrac distance revealed that samples with different phenotypes could be clustered obviously into two groups and separated from each other in the first axis (Figure 5B). Non-metric multidimensional scaling (NMDS) also showed significant separation between the two groups, suggesting a significant variation in microbial communities (Figure 5B). A total of 23 significantly altered genus were detected based on Linear discriminant analysis Effect Size (LEfSE) analysis with a threshold of log10 (linear discriminant analysis, LDA) > 2 and *p* < 0.05, including Butyricimonas, Parabacteroides, Mucispirillum, Desulfovibrio, etc. (Figure 6).

### 2.5. Correlation Analysis

**Figure 7:**
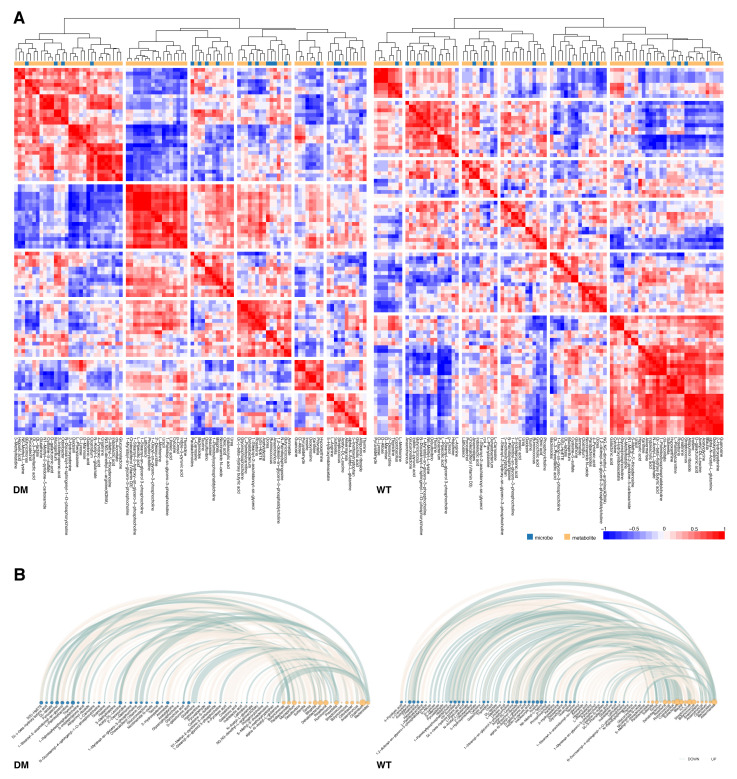
Correlation analysis of metabolites and microbes. (**A**) Heatmaps for correlation coefficients of metabolites and microbes in db/db and wt mice. Red represents positive correlation and blue represents negative correlation. (**B**) Arc diagrams for significantly correlated metabolite–microbe combinations. Light yellow edges represent significant positive correlations and light green edges represent significant negative correlations. Blue and yellow nodes represent microbes and metabolites, respectively.

**Figure 8:**
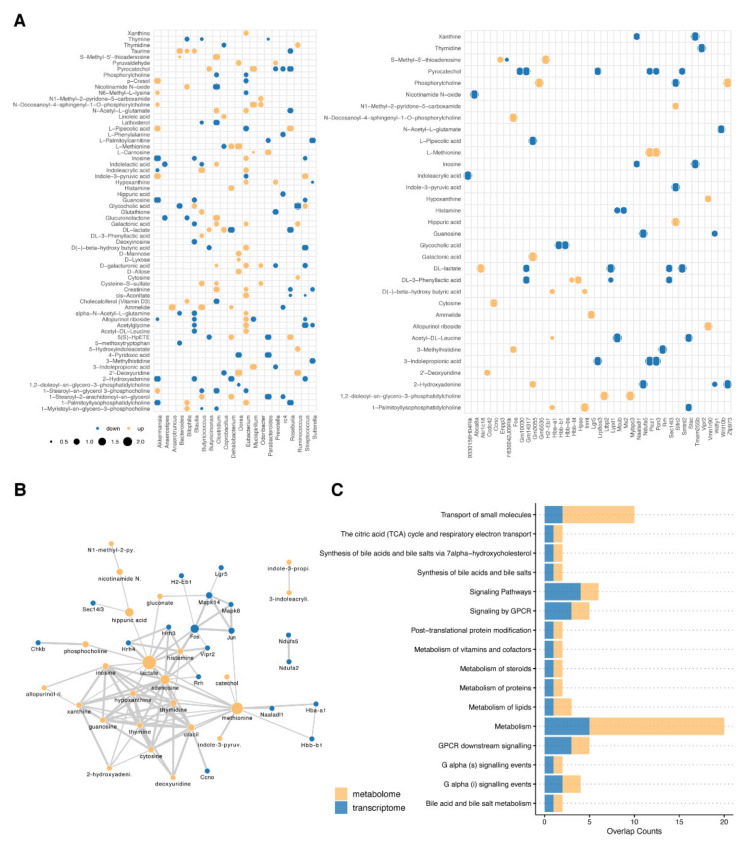
Integration of correlation analysis for metabolites–microbes and metabolites–genes. (**A**) Scatter plots for the alteration of correlation coefficients. Compared to wt mice, red represents an increase in the correlation coefficient, while blue represents a decrease. The size of the dots represents the absolute value of the change in correlation coefficients. (**B**) Interaction network of selected metabolites and genes based on STITCH database. The color of each node represents the classification of each selected feature. The size of the node represents the betweenness centrality. The thickness of each edge represents the interaction strengths between nodes. (**C**) Bar graph for Over-representation analysis (ORA) of selected metabolites and genes based on the Reactome database.

Spearman correlation analyses were carried out on 75 metabolites and 23 genera with significant alteration in db/db and wt mice, respectively. As shown in Figure 7A, the clustering of the two groups differed significantly. *p* < 0.05 and absolute values of correlation coefficients greater than 0.6 were set as the threshold to define significant correlations. A total of 82 metabolite–genus correlation pairs were detected in the db/db group, of which 49 were significantly positively correlated and 33 negatively correlated. In wt mice, 124 significantly metabolite–genus correlation pairs were detected, of which 74 were positively correlated and 50 were negatively correlated (Figure 7B). The correlation coefficients of 164 metabolite–genus correlation pairs consisting of 64 metabolites with 22 genera were drastically altered between db/db and wt groups (Figure 8A). Among them, in the db/db group, the correlations of 80 combinations were increased (Dorea—2′-Deoxyuridine, Dorea—L-Methionine, and Bilophila—1-Myristoyl-sn-glycerol-3-phosphocholine, etc.) and 84 were decreased (Sutterella—3-Methylhistidine, Bilophila—Glucuronolactone and Bilophila—Thymine, etc.).

Integration using linear models of metabolomics and gene expression data (IntLIM) was further used to detect the alteration of correlation between DEGs and metabolites in both db/db and wt mice. The absolute value of the change in correlation coefficient > 0.5 and *p* < 0.01 were defined as the threshold to filter the metabolite–gene correlations. Based on the metabolites list from the metabolite–genus combinations in the previous step, 68 pairs of metabolite–gene correlations (consisting of 33 metabolites and 41 genes) were screened as significantly altered (Figure 8A). According to the STITCH database, the interaction network between candidate genes and metabolites was constructed, which was enriched in “Synthesis of bile acids and bile salts via 7 alpha-hydroxycholesterol”, “Synthesis of bile acids and bile salts”, “Bile acid and bile salt metabolism”, “G alpha (i) signaling events”, “G Protein-Coupled Receptor (GPCR) downstream signaling”, “Signaling by GPCR”, “Metabolism of steroids”, “G alpha (s) signaling events”, “Metabolism of vitamins and cofactors”, “Metabolism”, “The citric acid (TCA) cycle and respiratory electron transport”, “Transport of small molecules”, “Signaling Pathways”, “Metabolism of lipids”, “Post-translational protein modification”, and “Metabolism of proteins” (Figure 8B,C).

### 2.6. Integrated Multi-Omics Analysis

**Figure 9:**
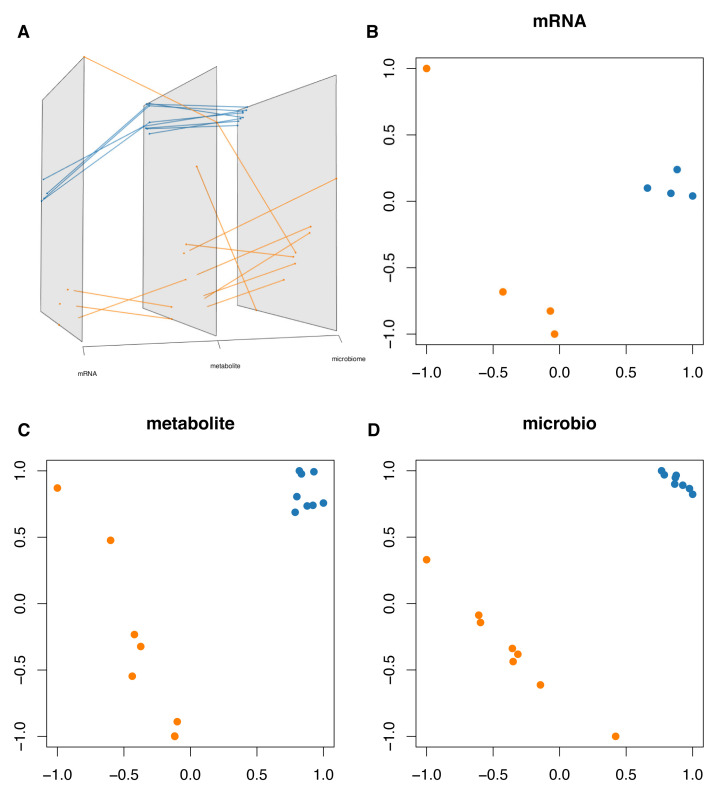
PLS-DA for multi-omics data. (**A**) Grimon visualization for multi-omics data. Each plane represents a two-dimensional visualization of the Partial least squares discriminant analysis (PLS-DA) result of each omics data. The same sample in different planes was connected. (**B**–**D**) Two-dimensional projections of PLS-DA results in each grimon plane. The color represents the group of each sample.

**Figure 10:**
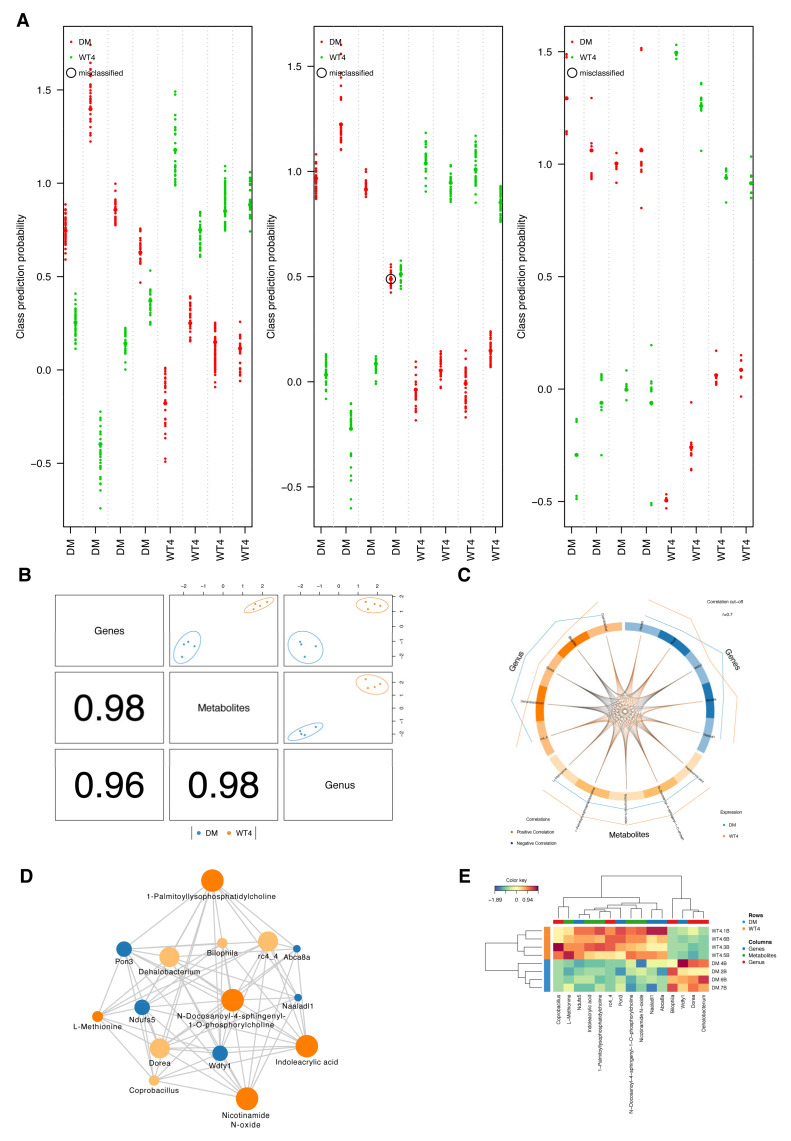
Performance of integrative modeling on multi-omics in db/db and WT mice. (**A**) Swim lane plots for classification analysis using multivariate methods with unbiased variable selection in R (MUVR). Each lane presents predictions for each sample, individually and overall. The colors of the dots represent the prediction of classes. The smaller dots indicate the class probabilities from individual repetitions, while the larger dots represent probabilities averaged over all repetitions. Circled dots represent the misclassified predictions. (**B**) The diagnostic plot indicates the correlation between components from each omics data and the power to separate two groups of mice. (**C**,**D**) The circos plot and relevance network represent correlations between selected signatures from each omics data. Orange and black edges of the circos plot indicate positive and negative correlations, respectively. The size of each node in the relevance network represents the betweenness centrality. (**E**) The heatmap represents the scaled expression of selected signatures from each omics data.

Partial least squares discriminant analysis (PLS-DA) was performed in each omics data, suggesting that db/db and wt mice were significantly separated (Figure 9). After integrating correlation pairs of metabolites–genus and metabolites–genes, a multivariate method with unbiased variable selection in R (MUVR) was carried out for candidate genes (*n* = 41), metabolites (*n* = 33) ,and genus (*n* = 21). The predictive accuracy of candidate omics signatures were100%, 87.5%, and 100%, respectively, indicating a significant separation between the two groups (Figure 10A).

Data integration analysis for biomarker discovery using a latent component method for omics (DIABLO) was further performed on the selected multi-omics signatures, and latent components composed of five features from each omics data were identified. The latent components were highly correlated to each other and contributed to a great separation between the two groups (Figure 10B). The close correlation between different types of omic signatures is shown in Figure 10C,D. Based on the betweenness centrality, Nicotinamide N-oxide, N-Docosanoyl-4-sphingenyl-1-O-phosphorylcholine, Indoleacrylic acid, and 1-Palmitoyllysophosphatidylcholine may play key roles in the network. Compared with wt mice, the expression or abundance of Coprobacillus, L-Methionine, Ndufs5, Indoleacrylic acid, 1-Palmitoyllysophosphatidylcholine, rc4_4, Pon3, N-Docosanoyl-4-sphingenyl-1-O-phosphorylcholine, Nicotinamide N-oxide, Naaladl1, and Abca8a were significantly decreased, while expression or abundance of Bilophila, Wdfy1, Dorea, and Dehalobacterium were significantly increased (Figure 10E).

## 3. Discussion

In the present study, we applied omics-based approaches to explore the core differentially expressed pathways and molecules underlying T2DM-associated cognitive deficits. Using the multi-omics integration, significant correlations among key genes (*n* = 33), metabolites (*n* = 41), and bacterial genera (*n* = 21) were identified under pathological state. Further functional analysis revealed that they are mainly involved in the disturbances of the TCA cycle, mitochondrial respiratory electron transport, bile acid synthesis and metabolism, steroid, and lipid metabolism and GPCR-related signals, etc. Taken together, our results provided an essential link between the brain–gut axis and cognitive impairment in db/db mice.

The mitochondrial metabolism-related molecules, especially those associated with oxidative phosphorylation and the TCA cycle were differentially expressed in both brain genes and serum metabolites. Those genes, such as Ndufs5 (encoding NADH dehydrogenase, complex I), COX3 (encoding cytochrome C oxidase III), Me2 (encoding malic enzyme 2), and Atp5d (encoding ATP synthase F1 subunit), showed lower expression levels in db/db mice compared to wt mice. NADH (Nicotinamide adenine dinucleotide) dehydrogenase as the first enzyme complex in the respiratory electron chain, functions in transferring electrons derived from NADH to oxygen molecules [28]. Likewise, cytochrome C oxidase is the rate-limiting enzyme at the end of the mitochondrial respiratory chain, also known as mitochondrial complex IV [29]. By converting oxygen molecules into H_2_O and driving ATP synthase to produce ATP, complex IV plays an important role in cell energy metabolism [29]. Impairment of the respiratory chain will lead to a decrease in ATP synthesis and excessive production of reactive oxygen species (ROS), which in turn induces cell apoptosis [30]. It has been proposed that high concentrations of ROS can promote the accumulation of hypoxia-inducible factors (HIF-1), and the cumulative effect of HIF-1 leads to a decrease in the TCA cycle as well as an increase in anaerobic glycolysis [31]. Similarly, the HIF-1 signaling pathway was upregulated in the transcriptome analysis of this study. Consistent with alterations in brain transcription, the intermediate products of the TCA cycle, malic acid, showed a cumulative increase in serum metabolites, while the products of anaerobic glycolysis, lactate were increased significantly. Sickmann et al. discovered that the TCA cycle activity was reduced more than anaerobic glycolysis in the brain of T2DM rats [32]. Lactate functions as a signaling molecule that not only regulates the polarization and inflammatory response of macrophages, but also induces the release of TNF-α, IL-6, and IL-8 from astrocytes cultured in vitro [33]. Previous studies also have found that an increased lactate concentration may be linked to cognitive deficits in human and animal models [34,35,36]. Thus, our hypothesis-free approach further supported that the disturbances of circulated and central energy metabolism may contribute to cognitive impairment in db/db mice.

We also observed disturbances of bile acid and steroid metabolism in both transcriptome and metabolome analysis, which provide evidence that the close interaction among microbiota, metabolites, and brain may play a key role in db/db mice with cognitive deficits. Hepatocytes are the primary site of bile acid synthesis in humans and mice. Prior to secretion from the liver, primary bile acids are combined with glycine or taurine to generate conjugated bile acids, such as glycocholic acid, which are subsequently secreted into the gallbladder for storage with phospholipids and cholesterol [37,38]. Conjugated bile acids are thereafter released into the intestine and further metabolized by intestinal microorganisms into secondary bile acids, such as deoxycholic acid [37,38]. It has been considered that bile acids modulated by the gut microbiota facilitate absorption and digestion of lipids and fat-soluble vitamins [38]. Importantly, bile acids can act as signaling molecules involved in the regulation of brain function, and the majority of brain bile acids derive from systemic circulation [39]. Our results showed that the levels of taurine and cholesterol were significantly increased, and the Slc10a1 gene (encoding taurocholate co-transporting polypeptide) was significantly downregulated, both of which indicated impaired bile acid transport. Studies verified that taurine concentration was increased in the brain of whether aged diabetic rats or db/db mice with cognitive decline or early AD rats [40]. Clinical investigations also suggested that elevated taurine levels may indicate the occurrence of early cognitive impairment, and its level is negatively correlated with the progress of cognitive impairment [40,41]. Therefore, it is possible that bile acids as a potential link between the gut microbiome and the brain play a critical role in cognitive impairment.

The downregulation of GPCR signaling further supported the relative interactions between brain and gut microbiota. GPCR, the G protein-coupled receptor superfamily, comprises various receptors, such as glucagon-like peptide 1 (GLP-1) receptor, free fatty acid receptor 1 (GPR40), and G-protein-coupled bile acid receptor 1 (Gpbar1), which are involved in the regulation of neurotransmitters, hormones, nutrients, and metabolites, etc. [42]. Studies by Kristoffer and colleagues have revealed that intestinal-derived molecules may modulate the vagal afferents present in the gastrointestinal mucosa via GPCR signaling, and thereby, signaling to the brain [43]. Under physiological conditions, Gpbar1 signaling contributes to the release of intestinal GLP-1, and circulating GLP-1 further activates GLP-1 receptors in intestinal vagal afferents to indirectly influence the metabolism of brain glucose and bile acid [44,45]. More recent, striking evidence demonstrates the presence of Gpbar1 in the astrocytes and neurons suggesting this gut-derived signaling may directly affect brain function [46,47]. In the AD mice, activation of Gpbar1 with tauroursodeoxycholic acid (TUDCA) triggers the AKT/GSK3β pathway to exert anti-inflammatory effects [48,49]. Additionally, induction of TUDCA can inhibit mitochondrial apoptosis through the E2F1/p53/BAX pathway to reduce neuronal death as well as Aβ deposition [50,51]. In T2DM mouse models, researchers have found that downregulation of GPR40 correlates with decreased expression of brain-derived neurotrophic factor (BDNF), which might be an underlying molecular mechanism associated with cognitive impairment in diabesity [52]. Collectively, these findings further support that GPCR signaling is critical to regulating normal brain function, becoming a potential therapeutic target for cognitive disabilities.

Furthermore, through multi-omics analysis, significantly correlated key molecules were identified as potential biomarkers, which may predict the disease status of mice. NADH dehydrogenase, the entry enzyme for mitochondrial oxidative phosphorylation, was encoded by Ndufs5. Downregulation of this gene may indicate mitochondrial dysfunction and developing oxidative stress [53]. Paraoxonase 3, encoded by Pon3, has antioxidative and anti-inflammatory properties, as well as a protective effect on mitochondria by regulating superoxide production and resisting apoptosis [54]. The Naaladl1 gene encoding a homolog of glutamate carboxypeptidase II is widely expressed in the small intestine. This metallopeptidase could function as imaging and therapeutic targets for neuronal injury [55]. ATP binding cassette subfamily A member 8 was encoded by Abca8. Apart from the promotion of lipid transport in the brain, the function of this protein in the central nervous system is still not well known [56], and further exploration of the role of Abca8 in neurodegenerative diseases is needed. The protein encoded by Wdfy1 is a phosphatidylinositol 3-phosphate binding protein. The upregulation of this protein would enhance the activation of NF-κB, type I interferon, and inflammatory cells through the TLR3 and TLR4 pathways, thereby promoting inflammation [57]. Consistent with our results, previous research has demonstrated that Bilophila, Akkermansia, and Dehalobacterium were positively correlated with bile acid (such as taurocholic acid) level, Coprobacillus positively associated with glucose metabolism, Dorea positively related to pyruvate level, rc4_4 positively correlated with tryptophan metabolism [58]. Methionine, one of the essential amino acids, as a major source of methyl groups is closely related to the folate cycle and the one-carbon cycle. Recent studies have shown that methionine deficiency could cause homocysteine metabolism disorder and mitochondrial dysfunction, and as a consequence lead to hypomethylation of choline metabolism in the hippocampus, as well as reduced choline levels and cognitive deficits [59,60]. Indoleacrylic acid is one of the products of tryptophan decomposition by intestinal microbiota, which is closely related to tryptophan metabolism and has the effects of enhancing intestinal epithelial barrier function, regulating glucose metabolism, and anti-inflammation [61]. Previous studies have also reported that indole propionic acid, another tryptophan metabolite, plays a potential neuroprotective role by scavenging oxygen free radicals to prevent Aβ-induced neuronal damage [62]. 1-Palmitoyllysophosphatidylcholine and N-docosanoyl-4-thienyl-1-O-phosphorylcholine are phospholipids, both of which are closely related to lipid metabolism. Niacinamide N-oxide belongs to the nicotinamides, a class of organic compounds, which can promote the production of NADP (+) and NADPH, playing a role in blood glucose control and neuroprotection [63].

Altogether, these findings reflected that the influence of the above key predictors on cognitive impairment in db/db mice should not be underestimated. However, correlation does not imply causation. In the future, collecting multi-omics data from mice at different time points and making causal inferences may be conducive to obtaining more specific conclusions. In addition, experiments are still needed to further verify and explore the bioinformatics prediction results. This study provides novel insights into the functional interactions among the brain, circulating metabolites, and gut microbiota. Further functional investigations underlying the mechanisms behind the dysregulation of energy metabolism and cognitive decline will become a critical step in the development of future targeted therapies.

## 4. Materials and Methods

### 4.1. Animals

Fourteen-week-old diabetic (db/db, B6.BKS(D)-Lepr^db/db^, 4 males and 4 females) and wt (C57BL/6J, 4 males and 4 females) mice were purchased from the Shanghai Model Organisms Center, Inc. (Shanghai, China). Genotype, body weight, and fasting blood glucose were assessed by the Shanghai Model Organisms Center, Inc. (Shanghai, China). The animals were fed in a specific pathogen-free environment under the standard conditions (12:12-h light-dark cycle, 22 ± 2 °C room temperature, and a relative humidity of 40–60%). All animals were given unlimited access to standard sterile chow and tap water during the whole experimental period. All animal experiments were conducted based on the Guide for the Care and Use Laboratory Animals of the Ruijin Hospital. This study was reviewed and approved by the Institutional Animal Care and Use Committee of the Shanghai Jiao Tong University.

### 4.2. Morris Water Maze Test

After 3 weeks, the MWM test was performed to assess the spatial learning and memory of the mice. The MWM test was conducted in a circular pool (diameter 100 cm, height 60 cm) filled with water at 22 ± 2 °C. On day 0, the mice were subject to habituation training with visible escape platform (1 cm above the water surface) and undyed water. Then the water was made opaque by food-grade titanium dioxide and the escape platform was submerged 2 cm below the water surface. The mice were trained to reach the hidden escape platform for 5 consecutive days (day 1–day 5, 4 trials per day). If a mouse could not find the escape platform within the maximum observation period (60 s), it was guided to the platform by the operator and allowed to stay for 10 s. On day 6, the mice were tested in a 60 s probe trial without the platform. For each trial, the time and latency to reach the platform and the number of platform crossings were recorded using EthoVision XT software (Noldus Information Technology b.v., Wageningen, The Netherlands). Analyses of the behavioral data were conducted using two-way ANOVA and *t*-test by Graphpad Prism 8.0 (GraphPad Software Inc., San Diego, CA, USA).

### 4.3. Sample Preparation

Before the MWM tests, fecal samples were collected about 100 mg (wet weight) per mouse after defecation, respectively. Extraction of microbial DNA was performed using the E.Z.N.A.^®^ Stool DNA Kit (Omega Bio-Tek, D4015-01, Norcross, GA, USA). After detecting the concentration and quality, the DNA was diluted to 1 ng/μL using sterilized water and stored at −80 °C prior to 16S rDNA sequencing.

After the MWM tests, retro-orbital sinus sampling of each anesthetized mouse was collected and processed to serum and stored at −80 °C before untargeted metabolomic analysis. Afterward, 100 μL serum was added to a 400 μL precooled methanol/ acetonitrile mixture (1:1), following vortex oscillation for 60 s. The mixture was placed at −20 °C for 30 min and centrifuged at 14,000× *g* for 20 min at 4 °C. Then, the supernatant was dried in a vacuum environment and dissolved by a 100 acetonitrile/water mixture (1:1), followed by vortex oscillation. After centrifugation at 14,000× *g* for 20 min at 4 °C, the upper phase as the sample was subjected to mass spectrometry analysis.

After general anesthesia, the mice were killed by cervical dislocation and brain tissues were isolated immediately. Total RNA was extracted from brain tissues using TRIzol Reagent (Thermo Fisher Scientific, 15596018, Waltham, MA, USA), according to the manufacturer’s protocol. Nanodrop ND-2000 system (Thermo Scientific, Waltham, MA, USA) was used to detect the A260/A280 absorbance ratio of RNA samples, and the Agilent Bioanalyzer 4150 system (Agilent Technologies, Santa Clara, CA, USA) was used to determine the RIN value of RNA. The qualified RNA was used for subsequent transcriptomic analysis.

### 4.4. Transcriptomic Analysis of Brain Samples

Library preparation and transcriptome sequencing were performed at the Shanghai Applied Protein Technology Co., Ltd. (APTBIO, Shanghai, China). In brief, a total of 1 μg RNA per sample was used for library construction by a NEBNext^®^Ultra™ RNA Library Prep Kit for Illumina (New England Biolabs, Ipswich, MA, USA), following the manufacturer’s protocol. An Agilent Bioanalyzer 2100 system (Agilent Technologies, Santa Clara, CA, USA) was used for library quality assessment. Paired-end reads were generated by the Illumina Novaseq 6000 platform (Illumina, San Diego, CA, USA). Raw data were processed through in-house perl scripts of APTBIO to remove the adapter sequence and filter out reads with low quality or an N ratio (the base information could not be determined) greater than 5%. Subsequently, clean data were aligned to the reference genome of mice using HISAT2, and reads numbers mapped to each gene were counted by FeatureCounts [64,65].

The EdgeR package was used for differential expression analysis [66]. *p* < 0.05 and the absolute value of log2 fold change (log2FC) > 1 were set as the threshold. To explore whether predefined gene sets were differentially expressed in different groups, we further performed GSEA using the multiGSEA package on the basis of the Reactome database [67,68]. Gene sets with *p* < 0.05 and the absolute value of normalized enrichment score (NES) > 1 were considered as significantly differentially expressed gene sets.

### 4.5. Metabolomic Analysis of Serum Samples

Liquid Chromatograph Triple Quadrupole Mass Spectrometer (LC-MS/MS) was carried out in APTBIO. Briefly, HILIC separation was performed using an Agilent 1290 infinity LC UHPLC system (Agilent Technologies, Santa Clara, CA, USA) and a 2.1 mm × 100 mm ACQUIY UPLC BEH 1.7 µm column (Waters Chromatography Ireland Ltd, Finglas, Ireland). The identification of metabolites and collection of primary and secondary spectra were performed using a TripleTOF 6600 System (SCIEX, Framingham, MA, USA). Raw data were converted to MzXML files by ProteoWizard before importing into XCMS software (Scripps Research Institute, La Jolla, USA) for peak picking, retention time correction, and peak area extraction [69,70]. Metabolites were identified using an in-house database based on the accuracy mass (<25 ppm) and MS/MS spectra.

After the Pareto-scaling using MetaboAnalyst, OPLS-DA was performed [71]. Combined with the results of a two-tailed Student’s *t*-test, metabolites with variable influence on projection (VIP) > 1 and *p* < 0.1 were considered as significantly different metabolites. MSEA was further carried out using the multiGSEA package on the basis of the Reactome database. *p* < 0.05 and the absolute value of NES > 1 were set as cutoff values [67,68]. The differences in the abundance of metabolites between the two groups were further detected by a Student’s *t*-test.

### 4.6. Fecal Microbiota Analysis

The amplification of 16S rRNA genes, library preparation, and sequencing were carried out in APTBIO. Specific primer with barcode (341F: CCTAYGGGRBGCASCAG, 806R: GGACTACNNGGGTATCTAAT) and Phusion^®^High-Fidelity PCR Master Mix (New England Biolabs, Ipswich, MA, USA) were used for amplification. The library was constructed using the NEB Next^®^Ultra™ DNA Library Prep Kit for Illumina (New England Biolabs, Ipswich, MA, USA), following the manufacturer’s protocol. A Qubit 2.0 Fluorometer (Thermo Fisher Scientific, Waltham, MA, USA) and an Agilent Bioanalyzer 2100 system (Agilent Technologies, Santa Clara, CA, USA) were utilized to assess the library quality. Paired-end reads were generated by the Illumina Novaseq 6000 platform (Illumina, San Diego, CA, USA) and merged using FLASH software (McKusick-Nathans Institute of Genetic Medicine, Baltimore, MD, USA) [72]. The UPARSE package was used for sequences analysis [73]. Sequences with at least 97% similarity were clustered as the same OTUs. Representative sequences for each OTU were annotated by the Ribosomal Database Project (RDP) classifier [74]. In-house perl scripts of APTBIO were run to analyze alpha and beta diversity. LEfSe was used to detect different features between two groups [75]. LDA > 2 and *p* < 0.05 were set as cutoff values to define significantly different genus.

### 4.7. Correlation Analysis

Spearman correlation analysis for significantly differential genes and genus in wt and db/db group were performed using the Hmisc package. With a correlation coefficient > 0.6 and *p* < 0.05 as the threshold values, the significantly correlated metabolites, and genus were defined. The absolute value of the differences between the correlation coefficients of the two groups greater than 0.5 was considered as drastically altered.

IntLIM can integrate transcriptome and metabolome data through linear models, to assess whether the association relationship between genes and metabolites was significantly different between the two different phenotypes [76]. IntLIM analysis based on the DEGs, and differential metabolites was performed on the two groups, respectively. *p* < 0.01 and the absolute change in correlation coefficient > 0.5 were defined as the threshold value of the significantly changed correlation of metabolites–genes.

The lists of candidate genes, metabolites, and genus were obtained by integrating metabolites–genus with drastically altered correlation coefficients and metabolites–genes with significantly changed correlation coefficients. The STITCH database was used to construct the network of interaction between candidate protein–protein and protein–metabolites (minimum required interaction score = 0.4) [77]. Over-representation analysis (ORA) of candidate genes and metabolites was further performed by the fgsea package based on the Reactome database [78]. Cytoscape, ggplot2, pheatmap, and the ggraph package were used for data visualization [79,80,81,82].

### 4.8. Integrated Multi-Omics Analysis

PLS-DA was carried out in each omics data using ropls and visualized by the grimon package [83,84]. Multivariate predictive modeling on candidate genes, metabolites, and genus was constructed using MUVR based on partial least squares (PLS), respectively [85]. DIABLO was utilized to integrate multi-omics data from the same biological sample, which could maximize the common or relevant information among different omics while identifying key variables of each omics and grouping information [86]. The number of components was determined by MUVR. A tuning procedure was used to select the optimal number of key features in each omics.

## Figures and Tables

**Table 1:** Gene set enrichment analysis based on the Reactome database (top 50 based on *p*-value).

Pathway Name	*p*-Value	NES	Size
Gene expression (Transcription)	3.42 × 10^−19^	1.909039	916
Metabolism of RNA	3.14 × 10^−18^	2.090669	526
RNA Polymerase II Transcription	2.50 × 10^−17^	1.883699	820
Processing of Capped Intron-Containing Pre-mRNA	3.31 × 10^−13^	2.227814	223
Cell Cycle	4.68 × 10^−12^	1.87915	503
Generic Transcription Pathway	1.19 × 10^−11^	1.750073	705
mRNA Splicing	4.21 × 10^−11^	2.190093	174
pre-mRNA splicing	1.59 × 10^−10^	2.178913	168
Cell Cycle, Mitotic	3.26 × 10^−10^	1.870694	423
Cell Cycle Checkpoints	1.22 × 10^−7^	1.903855	230
M Phase	2.68 × 10^−7^	1.754935	315
S Phase	5.32 × 10^−7^	1.958874	131
G1/S Transition	1.52 × 10^−6^	2.018423	99
Extracellular matrix organization	4.83 × 10^−6^	−2.067058	185
Respiratory electron transport	6.76 × 10^−6^	−2.37502	85
Mitotic G1 phase and G1/S transition	6.94 × 10^−6^	1.900258	121
Metabolism	1.24 × 10^−5^	−1.39958	1391
Mitotic Metaphase and Anaphase	1.68 × 10^−5^	1.707057	199
Mitotic Anaphase	1.95 × 10^−5^	1.701565	198
DNA Replication	2.58 × 10^−5^	1.851959	114
Transport of Mature mRNA derived from an Intron-Containing Transcript	2.91 × 10^−5^	1.982446	67
DNA Repair	5.03 × 10^−5^	1.643199	240
Synthesis of DNA	6.43 × 10^−5^	1.816823	106
SUMOylation	6.54 × 10^−5^	1.731646	138
Transport of Mature Transcript to Cytoplasm	7.11 × 10^−5^	1.927207	75
DNA Replication Pre-Initiation	8.43 × 10^−5^	1.856579	77
G1/S DNA Damage Checkpoints	8.51 × 10^−5^	1.900279	63
G2/M Checkpoints	8.74 × 10^−5^	1.780989	121
Complex I biogenesis	9.29 × 10^−5^	−2.392202	52
RNA Polymerase II Transcription Termination	9.81 × 10^−5^	1.912934	60
Separation of Sister Chromatids	0.000126	1.702185	152
SUMO E3 ligases SUMOylate target proteins	0.000133	1.716686	133
Mitotic Prometaphase	0.000142	1.679872	163
Epigenetic regulation of gene expression	0.000157	1.997729	47
RNA Polymerase II Pre-transcription Events	0.00021	1.855255	75
The citric acid (TCA) cycle and respiratory electron transport	0.000253	−1.974008	156
Respiratory electron transport, ATP synthesis by chemiosmotic coupling, and heat production by uncoupling proteins.	0.000285	−2.125326	109
p53-Dependent G1 DNA Damage Response	0.000305	1.921084	61
p53-Dependent G1/S DNA damage checkpoint	0.000305	1.921084	61
Syndecan interactions	0.000318	−2.137732	14
Chondroitin sulfate biosynthesis	0.00032	−2.018888	18
ECM proteoglycans	0.000339	−1.985636	22
mRNA 3′-end processing	0.00035	1.874325	51
Transcriptional Regulation by TP53	0.000365	1.513781	268
DNA Double-Strand Break Repair	0.000366	1.682974	114
TP53 Regulates Transcription of Cell Cycle Genes	0.00044	1.955699	29
Non-integrin membrane-ECM interactions	0.000441	−1.994996	18
Cross-presentation of soluble exogenous antigens (endosomes)	0.000448	1.858045	41
MET activates PTK2 signaling	0.000453	−2.076443	14
Cellular responses to external stimuli	0.000508	1.458568	323

NES = normalized enrichment score; SUMO = small ubiquitin-related modifier protein; ECM = extracellular matrix; MET = mesenchymal–epithelial transition factor; PTK = protein tyrosine kinase.

**Table 2:** Metabolite set enrichment analysis based on the Reactome database.

Pathway Name	*p*-Value	NES	Size
Adaptive Immune System	0.039127	−1.399622	2
Aflatoxin activation and detoxification	0.046795	1.316706	1
Aspartate and asparagine metabolism	0.033776	1.490742	4
Biosynthesis of DHA-derived sulfido conjugates	0.046795	1.316706	1
Biosynthesis of maresin conjugates in tissue regeneration (MCTR)	0.046795	1.316706	1
Biosynthesis of protectin and resolvin conjugates in tissue regeneration (PCTR and RCTR)	0.046795	1.316706	1
C-type lectin receptors (CLRs)	0.039127	−1.399622	2
CLEC7A (Dectin-1) signaling	0.039127	−1.399622	2
Cytokine Signaling in Immune system	0.007981	−1.639997	4
DAG and IP3 signaling	0.039127	−1.399622	2
ERK1/ERK2 pathway	0.014242	−1.575384	3
Fc epsilon receptor (FCERI) mediated NF-κB activation	0.039127	−1.399622	2
FLT3 Signaling	0.007981	−1.639997	4
FCERI signaling	0.039127	−1.399622	2
Gamma carboxylation, hypusine formation, and arylsulfatase activation	0.034649	−1.505648	4
Glycerophospholipid biosynthesis	0.027534	1.563377	11
Histidine catabolism	0.018443	−1.610865	5
MAPK family signaling cascades	0.014242	−1.575384	3
Metabolism	0.015173	−1.465771	109
Nucleobase catabolism	0.004696	−1.825806	22
Nucleotide metabolism	0.007471	−1.804005	24
Nucleotide salvage	0.000174	−2.036357	17
Phenylalanine and tyrosine metabolism	0.009249	1.590928	4
Purine salvage	0.001607	−1.851134	8
Pyrimidine catabolism	0.014483	−1.661637	11
Pyrimidine salvage	0.004541	−1.800858	9
Pyruvate metabolism	0.019494	1.606548	5
Pyruvate metabolism and Citric Acid (TCA) cycle	0.012356	1.706404	6
RAF/MAP kinase cascade	0.014242	−1.575384	3
RAS processing	0.014242	−1.575384	3
SLC-mediated transmembrane transport	0.036751	−1.512534	40
Synthesis of diphthamide-EEF2	0.034649	−1.505648	4
The citric acid (TCA) cycle and respiratory electron transport	0.004119	1.757392	7
Transport of nucleosides and free purine and pyrimidine bases across the plasma membrane	0.002303	−1.831755	11
Transport of small molecules	0.025099	−1.574603	45
Transport of vitamins, nucleosides, and related molecules	0.010163	−1.765573	24

NES = normalized enrichment score; DAG = diacylgycerol; IP = inositol triphosphate; ERK = extracellular signal-regulated kinase; NF-κB = nuclear factor κB; FLT = fms-like tyrosine kinase; MAPK = mitogen-activated protein kinase; RAF = rapidly accelerated fibrosarcoma; MAP = mitogen-activated protein; RAS = rat sarcoma virus; SLC = solute carrier; EEF = Eukaryotic elongation factor.

## Data Availability

The data presented in this study are available on request from the corresponding author.

## Supplementary Materials

The following supporting information can be downloaded at https://www.mdpi.com/article/10.3390/molecules27061904/s1, Table S1: Gene count of mice, Table S2: Metabolites in negative mode, Table S3: Metabolites in positive mode, Table S4: OTUs of mice.

## Sample Availability

Not available.
